# Announcement of two complete coding genomes of mink coronavirus and one partial coding genome of mink enteric calicivirus from mink in Denmark

**DOI:** 10.1128/mra.00459-25

**Published:** 2025-11-12

**Authors:** Christina M. Lazov, Lars E. Larsen, Camille M. Johnston, Thomas Bruun Rasmussen, Charlotte K. Hjulsager

**Affiliations:** 1 Department of Virology and Microbiological Preparedness, Statens Serum Institut 4326, Copenhagen, Denmark; 2 National Veterinary Institute, Technical University of Denmark, Kongens Lyngby, Denmark; 3 Department of Veterinary and Animal Sciences, University of Copenhagen 87017, Frederiksberg, Denmark; Katholieke Universiteit Leuven, Leuven, Belgium

**Keywords:** mink coronavirus, sapovirus, metagenome, mink enteric calicivirus, diarrhea

## Abstract

Two complete coding genomes of mink coronavirus and one partial coding genome of the sapovirus mink enteric calicivirus were assembled from metagenomic sequencing data from mink on different farms with diarrhea outbreaks in 2015 in Denmark.

## ANNOUNCEMENT

Mink coronaviruses (MCoVs) belonging to the species *Alphacoronavirus neovisontis*, family *Coronaviridae* has previously been associated with gastrointestinal disorders in farmed mink (*Neogale vison*) (1). Few complete sequences of MCoV strains and even fewer and only partial genome sequences of mink enteric calicivirus (MEC), belonging to the genus *Sapovirus* (genotype GXII) in the *Caliciviridae* family, appear presently in GenBank. This virus has also been linked to disease, as it has been isolated from diarrheic mink (2), and the replication and location of mink sapovirus in the crypts and basis of the villi of the small intestine from diarrheic mink kits has been visualized by *in situ* hybridization (3).

This report describes the detection and sequencing of complete coding genomes of MCoV strains and a partial coding genome of MEC from two minks on different farms sampled in 2015 in Denmark.

Mink feces samples, from farms experiencing problems with diarrhea, were collected in July 2015. The samples were diluted in PBS to 10% and homogenized in a TissueLyser II (QIAGEN, Denmark). Nucleic acids were extracted using the QIAsymphony DSP Virus/Pathogen Mini Kit (QIAGEN, Denmark) on a QIAsymphony (QIAGEN, Denmark). The presence of coronavirus was tested with a broadly reactive panCoV RT-qPCR assay (4, 5). Two RNA samples testing positive for coronavirus were selected for sequencing using a non-targeted metagenomic protocol as previously described (6). Briefly, dsDNA was generated using the NEBNext mRNA second strand synthesis kit (New England Biolabs), library preparation performed with the Nextera-XT DNA library Preparation kit, and sequencing done on a MiSeq machine with Reagent Kit v3 600 bp (Illumina Inc, San Diego, CA, USA).

Taxonomic identification on read level was performed with Kaiju (7) (Table 1), read quality assessed using FastQC (8), and trimming performed with BBDuk (9) (default parameters) using Geneious Prime (10). Trimmed reads were *de novo* assembled using the SPAdes plugin v. 3.10.0 for metagenomic data (default parameters) (11). A single contig covering the complete coding genome of MCoV was generated for each of the two samples 11917-2 and 11918-1, as well as a single contig with the partial coding genome of MEC in sample 11917-2 (Table 1). Contigs were polished using trimmed reads, and consensus sequences were extracted after visual evaluation. These were queried by BLASTn, aligned to the highest scoring reference genomes, and annotated.

**TABLE 1:** Overview of samples, read data, and assemblies described in this study

Parameters	Data set	Data set	
Sample number	11918-1	11917-2	
Total reads	453,092	2,728,992	
Trimmed reads	420,000	2,300,000	
Read length (min.–max.)	35–301	35–301	
Relevant results from Kaiju analysis	Alphacoronavirus	Alphacoronavirus, Sapovirus	
Virus strain	MCoV1/11918-1/DK/2015	MCoV1/11917-2/DK/2015	MEC/11917-2/DK/2015
Trimmed reads mapped (approx. count)	85,600	460,000	1,700
Mean mapped read length (approx. bp)	180	170	160
Mean read coverage depth	546	2,800	37
Assembled genome length (bp)	28,914	28,910	7,327
GC content (%)	37.1	37.2	57.0
Closest BLASTn match (by % ID)	PQ451285, Mink coronavirus isolate MCoV/46351- 1_Case/DK/2019	PQ451290, Mink coronavirus isolate MCoV/46367- 5_Case/DK/2019	KX000385, Sapovirus GXII/WD1237
Identity to BLASTn match; E-value	91.94%; E 0.0	93.69%; E 0.0	83.60%; E 0.0 (78 % query coverage length)

The MCoV shared 91% nucleotide identity and 92% amino acid identity, indicating two different strains. Concerning the MEC, conserved motifs previously described for sapoviruses (12, 13) were used to roughly identify the different non-structural and structural viral protein CDS in the genome. Hereby, it was possible to determine that the 5′-end of ORF1 encoding the polyprotein and 3′-end of ORF2 encoding VP2 were missing. MEC reads identified in publicly available metagenomic data from farmed mink in Denmark (14) were used to confirm the MEC sequence assembly. Assembly and read information are shown in Table 1.

## Data Availability

The data and sequences have been deposited and have been publicly available in GenBank since June 2020 under the BioProject PRJNA577843, nucleotide sequences MN535737, MN535736, and MN543743.

## AFTER EPUB

*[This article was published on 12 November 2025 with errors in the third affiliation. The affiliation was corrected in the current version, posted on 28 November 2025.]*

## References

[1] Vlasova AN, Halpin R, Wang S, Ghedin E, Spiro DJ, Saif LJ. 2011. Molecular characterization of a new species in the genus Alphacoronavirus associated with mink epizootic catarrhal gastroenteritis. J Gen Virol 92:1369–1379. doi:10.1099/vir.0.025353-0 21346029 PMC3168282

[2] Guo M, Evermann JF, Saif LJ. 2001. Detection and molecular characterization of cultivable caliciviruses from clinically normal mink and enteric caliciviruses associated with diarrhea in mink. Arch Virol 146:479–493. doi:10.1007/s007050170157 11338385 PMC7086843

[3] Birch JM, Leijon M, Nielsen SS, Struve T, Jensen HE. 2021. Visualization of intestinal infections with astro- and sapovirus in mink (Neovison vison) kits by in situ hybridization. FEMS Microbes 2:xtab005. doi:10.1093/femsmc/xtab005 37334236 PMC10117860

[4] Escutenaire S, Mohamed N, Isaksson M, Thorén P, Klingeborn B, Belák S, Berg M, Blomberg J. 2007. SYBR Green real-time reverse transcription-polymerase chain reaction assay for the generic detection of coronaviruses. Arch Virol 152:41–58. doi:10.1007/s00705-006-0840-x 16941059 PMC7087200

[5] Lazov CM, Chriél M, Baagøe HJ, Fjederholt E, Deng Y, Kooi EA, Belsham GJ, Bøtner A, Rasmussen TB. 2018. Detection and characterization of distinct alphacoronaviruses in five different bat species in Denmark. Viruses 10:486. doi:10.3390/v10090486 30208582 PMC6163574

[6] Lazov CM, Belsham GJ, Bøtner A, Rasmussen TB. 2021. Full-genome sequences of Alphacoronaviruses and astroviruses from myotis and pipistrelle bats in Denmark. Viruses 13:1–20. doi:10.3390/v13061073 PMC8229204 34199948

[7] Menzel P, Ng KL, Krogh A. 2016. Fast and sensitive taxonomic classification for metagenomics with Kaiju. Nat Commun 7:11257. doi:10.1038/ncomms11257 27071849 PMC4833860

[8] Andrews S. 2018. FastQC v. 0.11.8. Babraham Institute Bioinformatics Group.

[9] Bushnell B. 2015. BBDuk Trimmer v. 1.0. Biomatters Ltd.

[10] Geneious Prime v. 2.3. 2019. Biomatters Ltd.

[11] Nurk S, Meleshko D, Korobeynikov A, Pevzner PA. 2017. metaSPAdes: a new versatile metagenomic assembler. Genome Res 27:824–834. doi:10.1101/gr.213959.116 28298430 PMC5411777

[12] Oka T, Lu Z, Phan T, Delwart EL, Saif LJ, Wang Q. 2016. Genetic characterization and classification of human and animal sapoviruses. PLoS One 11:e0156373. doi:10.1371/journal.pone.0156373 27228126 PMC4881899

[13] Oka T, Wang Q, Katayama K, Saif LJ. 2015. Comprehensive review of human sapoviruses. Clin Microbiol Rev 28:32–53. doi:10.1128/CMR.00011-14 25567221 PMC4284302

[14] Birch JM, Ullman K, Struve T, Agger JF, Hammer AS, Leijon M, Jensen HE. 2018. Investigation of the viral and bacterial microbiota in intestinal samples from mink (Neovison vison) with pre-weaning diarrhea syndrome using next generation sequencing. PLoS One 13:e0205890. doi:10.1371/journal.pone.0205890 30335814 PMC6193705

